# Butein induces cell apoptosis and inhibition of cyclooxygenase-2 expression in A549 lung cancer cells

**DOI:** 10.3892/mmr.2020.11616

**Published:** 2020-10-20

**Authors:** Yang Li, Chengyuan Ma, Ming Qian, Zhongmei Wen, Hongyu Jing, Donghua Qian

Mol Med Rep 9: 763-767, 2014; DOI: 10.3892/mmr.2013.1850

Following the publication of the above article, the authors have realized that an error was made in the Acknowledgements section in this paper; this research was not, in fact, supported by a grant from the National Natural Science Foundation of Jilin (Project no. 83657488), as had been stated.

The authors regret their oversight in providing this incorrect information in the Acknowledgements section of their paper. They thank the Editor of *Molecular Medicine Reports* for allowing them the opportunity to publish this corrigendum, and apologize to the readership of the Journal for any inconvenience caused.

